# Procoagulant genes may affect angiogenesis, epithelial-mesenchymal transition, survival prognosis and tumor immune microenvironment in patients with urothelial carcinoma

**DOI:** 10.18632/aging.204860

**Published:** 2023-07-08

**Authors:** Bin Li, Yuan Hu, Qiu-yang Li, Yi-Ming Tang, Zhe Lin

**Affiliations:** 1 Department of Urology, The First People’s Hospital of Foshan, Foshan, Guangdong, China; 2 Department of Obstetrics and Gynecology, The Third Affiliated Hospital, Sun Yat-sen University, Guangzhou, Guangdong, China; 3 Department of Urology, The Second Affiliated Hospital, Guangzhou Medical University, Guangzhou, China

**Keywords:** coagulation, bladder tumor, angiogenesis, EMT, immunosuppression

## Abstract

Factors related to coagulation regulation are closely related to angiogenesis, epithelial-mesenchymal transition, tumor proliferation and metastasis, and tumor immune microenvironment remodeling in tumors. To date, there are no quantitative indicators of coagulation associated with urothelial cancer. We classified urothelial cancer into high coagulation and low coagulation subtypes by screening for procoagulant-related molecular features and screened out relevant genes representing the coagulation state of urothelial carcinoma. Tumors with increased procoagulant gene expression were consistently associated with higher T-staging (*p* < 0.001), lymph node metastasis (*p* < 0.001), stage (*p* < 0.001), and grade (*p* = 0.046). Furthermore, high expression of procoagulant genes predicts a worse prognosis, a higher tumor proliferation rate and increased angiogenesis within the tumor. In addition, according to cibersort algorithm, the increased expression of procoagulant gene was negatively correlated with the degree of T-lymphocyte infiltration and positively correlated with the degree of M2 macrophage infiltration. Increased expression of procoagulant genes in data sets treated with immune checkpoints also predicted worse response and worse prognosis. At the same time, the expression of procoagulant genes in bladder cancer promoted the activation of coagulation, EMT, TGF-β and WNT pathways.

## INTRODUCTION

During the progression of advanced tumors, the body is often in a hypercoagulable state for various reasons. Cancer-related thrombosis is the leading cause of non-cancer mortality in malignancies [1]. In addition, coagulation-related factors also seem to influence the occurrence and development of tumors and the remodeling of microenvironment. Studies have confirmed that platelets can promote the distant metastasis of tumor cells by promoting epithelial-mesenchymal transformation of tumor cells [2]. In a research of colorectal cancer, the researchers found that an increase in fibrinogen levels was positively correlated with colorectal cancer as an independent risk factor [3]. Thrombin-related factors are closely related to tumor angiogenesis and tumor migration [4]. However, the current evidence mainly focuses on basic experiments and animal experiments, and has not been studied in clinical cohorts. In addition, the important role of coagulation-related factors in bladder cancer has not been reported yet.

It has been proved feasible to construct a risk signature with the expression of key genes to monitor the tumor and its microenvironment state [5–7]. We divided bladder cancer into two states of hypercoagulability and low coagulation by clustering coagulation-related genes, and we found that hypercoagulability presaged a poor prognosis of bladder tumors. Through further screening for coagulation-related differentially expressed genes and constructing risk signature, we found that hypercoagulable state is closely related to tumor angiogenesis, cancer cell proliferation, EMT transformation, and immune microenvironment remodeling.

## MATERIALS AND METHODS

### Patients and cohorts

The bladder cancer cohort from the Cancer Genome Atlas (TCGA) and GSE13057 database included information from 406 and 165 bladder cancer patients, respectively [8, 9]. The bladder cancer data set treated with PD1/PDL1 was derived from the Imvigor 210 data set and the GSE176307 data set for 298 and 88 patients, respectively [10, 11]. TCGA, GEO and Imvigor 210 are public databases in which patients have been ethically approved. Our research is based on open source data, so there are no ethical issues or other conflicts of interest. In our data screening process, cases with clinical information including survival state, survival time, clinical grade and stage, and complete information on genomic RNA sequence data met the inclusion criteria. Patients with incomplete information were removed. Data of the patients were downloaded through the Gene Expression Omnibus (GEO) database (https://www.ncbi.nlm.nih.gov/geo/) and TCGA database (https://portal.gdc.cancer.gov/repository). The data of Imvigor 210 was down were downloaded from (http://research-pub.gene.com/IMvigor210CoreBiologies). All genomic RNA sequence data were converted to transcripts per kilobase of exon model per million mapped reads (TPM), and standardize it by log2 [12].

### Consistent clustering of coagulation-related genes

We performed consistent clustering based on coagulation-related genes, and divided the bladder cohort of TCGA into hypercoagulable state and hypocoagulable state. We use K-Means algorithm to determine the state of the original data set and the number of queue clusters. This process is implemented through the R software package ConsensuClusterPlus [13], default parameters are adopted except that the number of clusters is set to 9. The correlation heat map was generated by R package “pheatmap” to observe the state distribution of each subtype.

### Screening of coagulation-related phenotypic genes and construction of coagulation-related risk signature

Differentially expressed genes (DEGs) between the distinguished subclasses according to coagulation status were screened by using R package limma [14]. DEGs between coagulation patterns were analyzed using empirical Bayesian statistics. Statistically significant genes were considered to meet the inter-group |log2-fold change (FC)|> 1 and error detection rate (FDR) < 0.05. The screening of key genes and the construction of coagulation-related models are realized by the latest absolute shrinkage and selection operator (LASSO) and multivariate COX regression [15–17]. Then the selected genes were input into COX regression model. After obtaining the coefficient corresponding to each gene and the intercept value in COX regression, the risk score of each sample is defined as coef1 × expression1 + coef2 × expression2+…, The specific classification ability of the model is reflected by receiver operating characteristic curve (ROC) and KM curve [18, 19]. After constructing the relevant risk model, we used the best critical value confirmed by X-tile software, and all bladder cancer queues were divided into high-risk and low-risk subgroups. The differences between OS\PFS in the two subgroups were compared by the practical logarithmic rank test of R package “survival”. R package “pROC” is used to visualize the distribution of corresponding risk values between the two groups. Using “pROC” package, the curve of ROC is obtained, and the prediction accuracy is judged by the area under the curve (AUC) value.

### Analysis of lymphocyte infiltration in tumor immune microenvironment

We calculated the infiltrating abundance of lymphocytes in each tumor sample using the cibersort deconvolution algorithm (CIBERSORT (https://cibersort.stanford.edu/) and its own LM22 lymphocyte genetic characteristics [20]. The obtained results are filtered (*p* < 0.05). The difference of infiltration between subgroups was evaluated by rank sum test, and the correlation between infiltration degree and risk score was evaluated by Pearson correlation.

### Functional and pathway enrichment analysis

Coagulation-related signature was analyzed using the Cluster Profiler software package for gene annotation enrichment analysis [21]. The Kyoto Encyclopedia of Genes and Genomes (KEGG) and the Gene Ontology (GO) functional enrichment analyses were carried out to clarify the biological function and behavior of coagulation-related signature. The GSEA was used to show the potential pathways and mechanisms between high-risk and low-risk subgroups.

### Statistical analysis

R software (R version 4.0.2 (2020-06-22)) was used for all analyses and graphics generated in this study. Inter-group comparisons were performed using the Kruskal-Wallis and *t* tests. If the normal distribution is satisfied, *t*-test is used. If the distribution is non-normal, Kruskal-Wallis test is used. Correlations were analyzed using the and Spearman correlation coefficient. Log-rank test, Cox regression and Kaplan-Meier curve were used to evaluate the prognostic value. All statistical analyses were bilateral, *P*-values < 0.05 were considered statistically significant.

## RESULTS

### Coagulation status of bladder cancer is closely related to survival prognosis and tumor microenvironment changes

**Figure 1:**
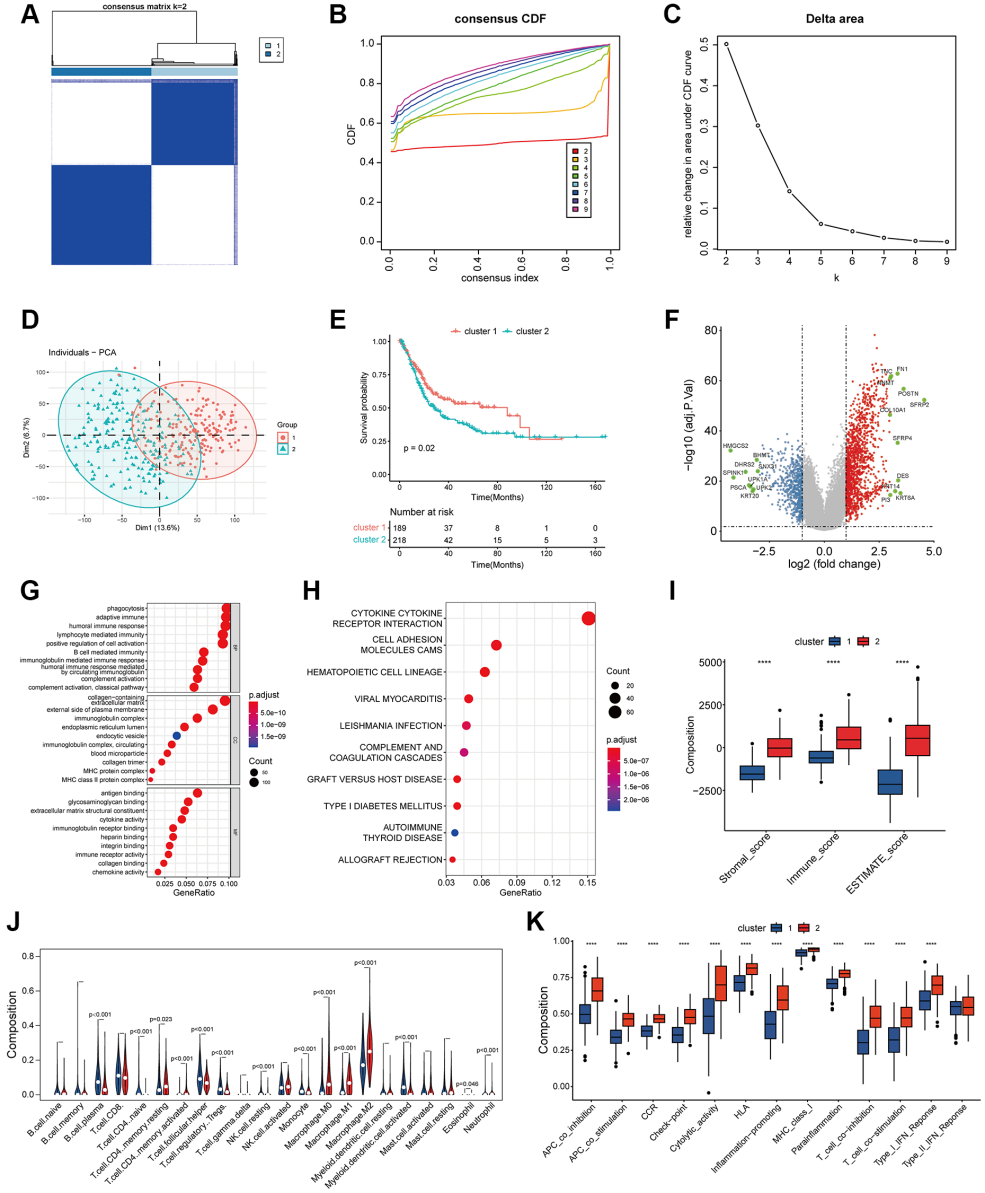
**Classification of coagulation subgroups in bladder cancer.** (**A**–**C**) The result of consensus matrix (**A**), consensus CDF (**B**) and delta area plot (**C**) suggested that the optimal value of K should be 2, which means it was most appropriate to divide all patients into two subtypes. (**D**) PCA classification of coagulation subtypes in bladder cancer. (**E**) K-M survival curve of coagulation subtypes in bladder cancer. The results suggest that hypercoagulable state of bladder cancer is significantly associated with poor prognosis. (**F**) Differentially expressed genes of coagulation subtypes in bladder cancer. Statistically significant genes were considered to meet the inter-group |log2-fold change (FC)|> 1 and error detection rate (FDR) < 0.05. (**G**) GO analysis suggested that biological function between coagulation subtypes was mainly enriched in the extracellular matrix, immune-related pathways, and complement and coagulation cascade pathways. (**H**) KEGG suggested that biological function between coagulation subtypes was mainly enriched in the extracellular matrix, immune-related pathways, and complement and coagulation cascade pathways. (**I**) The results of Estimate analysis suggest that hypercoagulable state has higher stromal score and estimate score. (**J**) Lymphocyte infiltration analysis of coagulation subtypes. The abundance of B cell and T cell infiltration with low coagulation state was higher, while the abundance of macrophage infiltration in the high coagulation state was relatively higher. (**K**) ssGSEA analysis of coagulation subtypes. APC-co-inhibition, check-point, HLA, MHC, T_cell and IFN displayed higher expression in the high coagulation state.

We divided bladder tumors into two subcategories according to the coagulation state by K-means clustering (Figure 1A–1D). At the same time, we analyzed the differences in survival outcomes between two different subclasses. The results suggest that hypercoagulable state of bladder cancer (cluster 2) is significantly associated with poor prognosis (*p* = 0.02) (Figure 1E). A total of 1792 significantly differentially expressed genes were screened, including 561 downregulated and 1231 upregulated (|log2FC| > 1, FDR < 0.05; Figure 1F). The results of GO and KEGG suggested that biological function was mainly enriched in the extracellular matrix, immune-related pathways, and complement and coagulation cascade pathways (Figure 1G, 1H). In addition, there were significant differences in the matrix, immune, and Estimate scores between the two subgroups (*p* < 0.001). In addition, the abundance of B cell and T cell infiltration in the immune microenvironment with low coagulation state was higher, while the abundance of macrophage infiltration in the high coagulation state was relatively higher (*p* < 0.001) (Figure 1H–1J). For immune-related pathways, the hypercoagulable state was also significantly different from the hypocoagulable state (*p* < 0.001) (Figure 1K). These results suggest that the coagulation state of the tumor plays an important role in various biological behaviors of the tumor and the composition of the tumor immune microenvironment.

### Establishment and verification of coagulation risk signature

**Figure 2:**
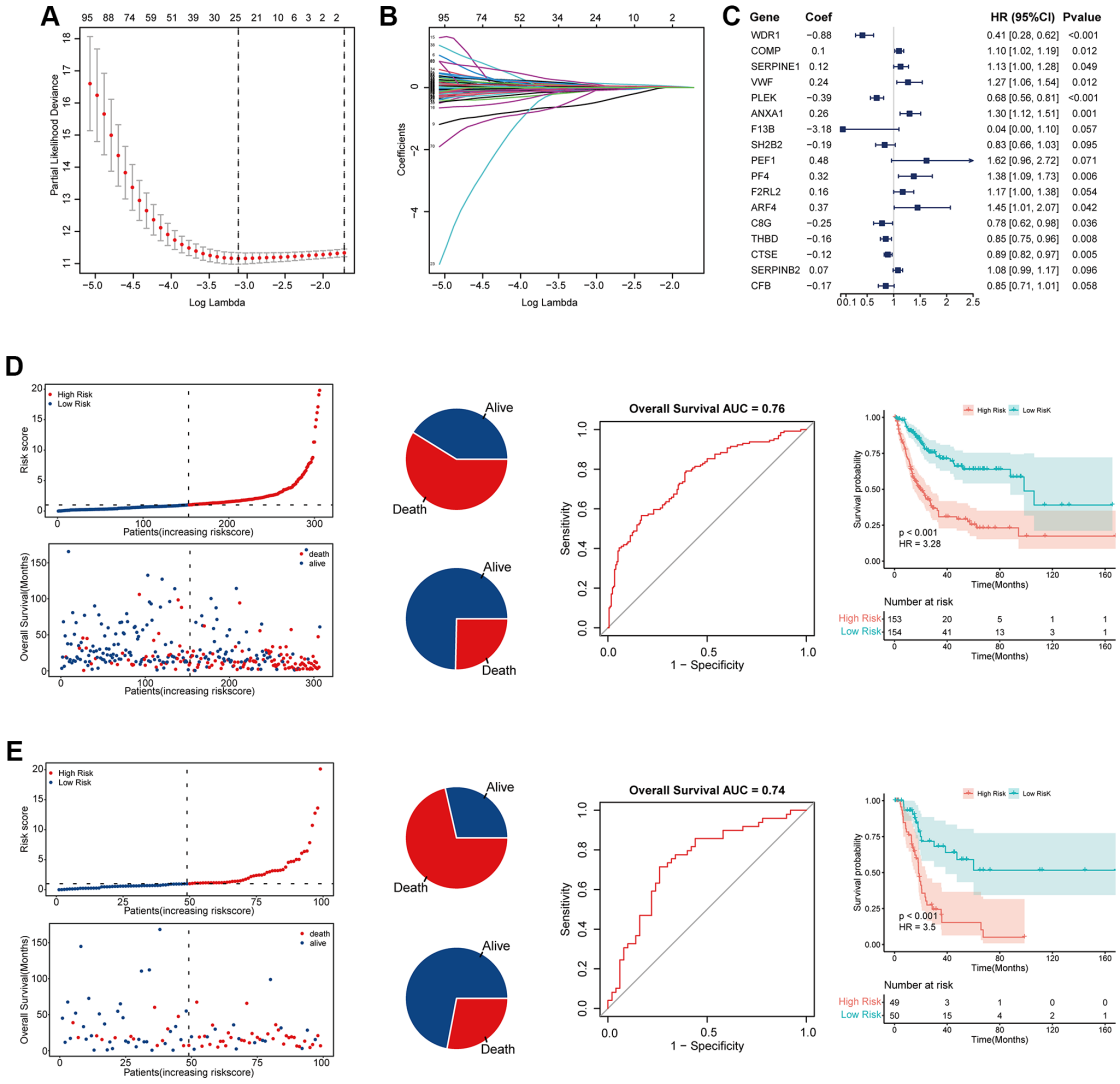
**Establishment and validation of coagulation-related risk signature.** (**A**) Least absolute shrinkage and selection operator cox analysis of the DEGs. (**B**) Partial likelihood deviance coefficient profiles. (**C**) Forest map of the variables contained in the risk signature. (**D**) Risk factor diagram, ROC curve and KM curve of the risk signature in the training set. The AUC of ROC curve is 0.76, while HR of KM curve is 3.28, *p* < 0.001. (**E**) Risk factor diagram, ROC curve and KM curve of the risk signature in the internal test set. The AUC of ROC curve is 0.74, while HR of KM curve is 3.5, *p* < 0.001.

**Figure 3:**
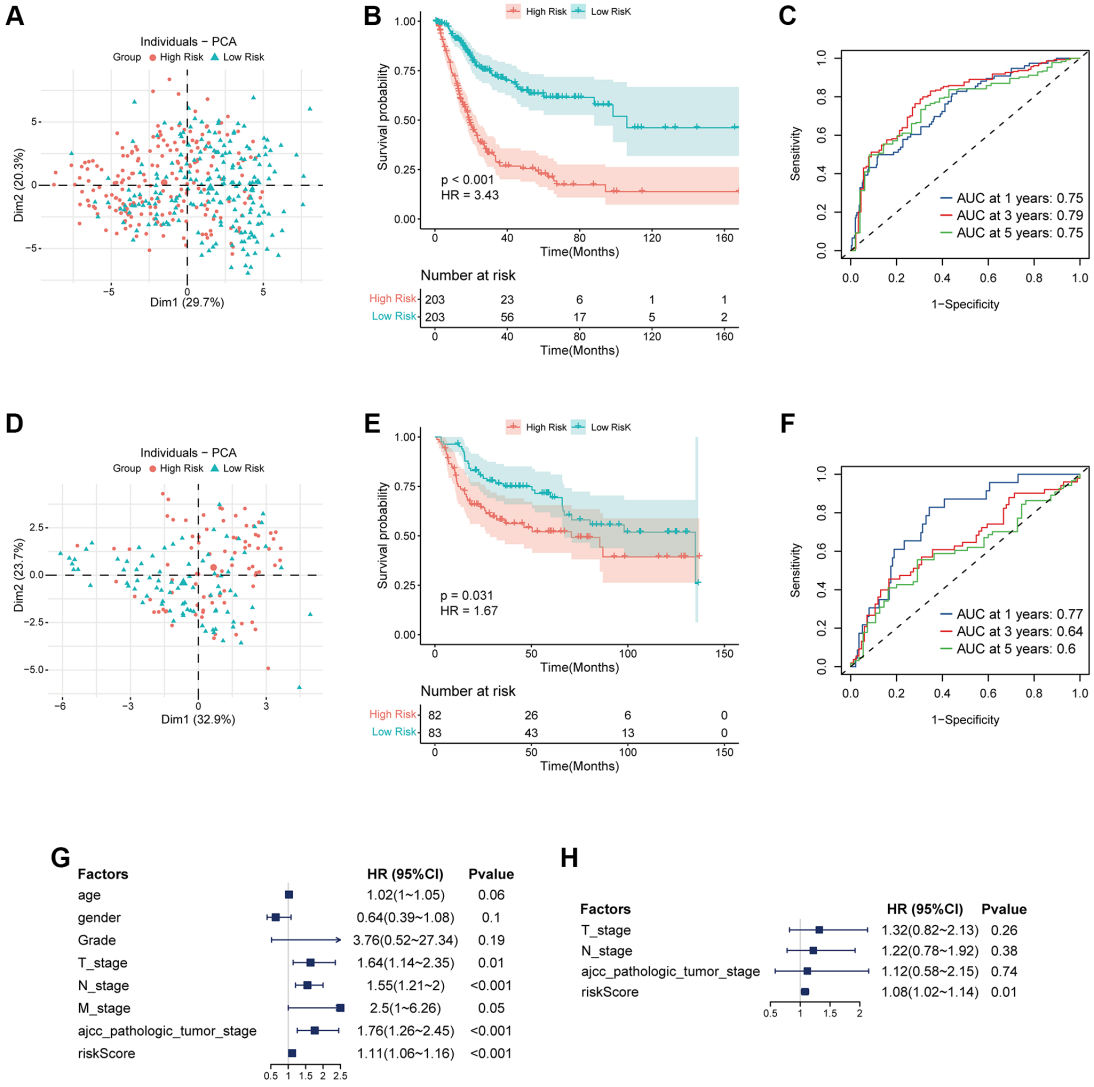
**Validation of coagulation-related risk signature in TCGA set and external validation set.** (**A**–**C**) PCA classification map, K-M curve, and ROC curve in TCGA dataset. The 1-year, 3-year, 5-year AUC of ROC curve is 0.75, 0.79, 0.75, respectively, while HR of KM curve is 3.43, *p* < 0.001. (**D**–**F**) PCA classification map, K-M curve, and ROC curve in external validation dataset. The 1-year, 3-year, 5-year AUC of ROC curve is 0.77, 0.64, 0.6, respectively, while HR of KM curve is 1.67, *p* = 0.031. (**G**) Multivariate COX regression forest map in TCGA dataset. Riskscore is an independent risk factor for prognosis. HR = 1.11, *p* < 0.001. (**H**) Multivariate COX regression forest map in external validation dataset. Riskscore is an independent risk factor for prognosis. HR = 1.08, *p* = 0.01.

We simultaneously constructed a coagulation-related risk signature by screening differentially expressed genes in different coagulation subtypes of bladder cancer and using the lasso-cox method (Figure 2A–2C). We split the TCGA data set into a training set and an internal verification set, and preliminarily verified the obtained risk signature. Our risk signature provides good predictability in both data sets. The area under the curve (AUC) in the training set was 0.76, and the KM curve suggested that the prognosis of patients in the high-risk and low-risk groups was significantly distinguished (HR = 3.28, *p* < 0.001). The AUC of the internal validation set was 0.74, and the prognosis of patients in the high-risk and low-risk groups was also well distinguished (HR = 3.5, *p* < 0.001). (Figure 2D, 2E). In order to further verify the sensitivity of our classifier, we made an in-depth analysis of the data of 165 bladder cancer patients in GSE13507 as an external validation set. In the overall data set of TCGA, the prognosis of patients in high and low risk groups is well differentiated (HR = 3.43, *p* < 0.001), and the AUC of one year, three years and five years are 0.75, 0.79 and 0.75 respectively (Figure 3A–3C). In the external validation set, the difference of prognosis between high and low risk groups was also statistically significant (HR = 1.67, *p* = 0.031), and the AUC of one year, three years and five years were 0.77, 0.64 and 0.6 respectively (Figure 3D–3F). Multivariate regression results suggested that our risk signature was an independent prognostic factor, whether in the TCGA dataset or in the external validation set (Figure 3G, 3H).

### The high risk associated with coagulation is closely related to tumor proliferation, angiogenesis, and immune microenvironment inhibition

**Figure 4:**
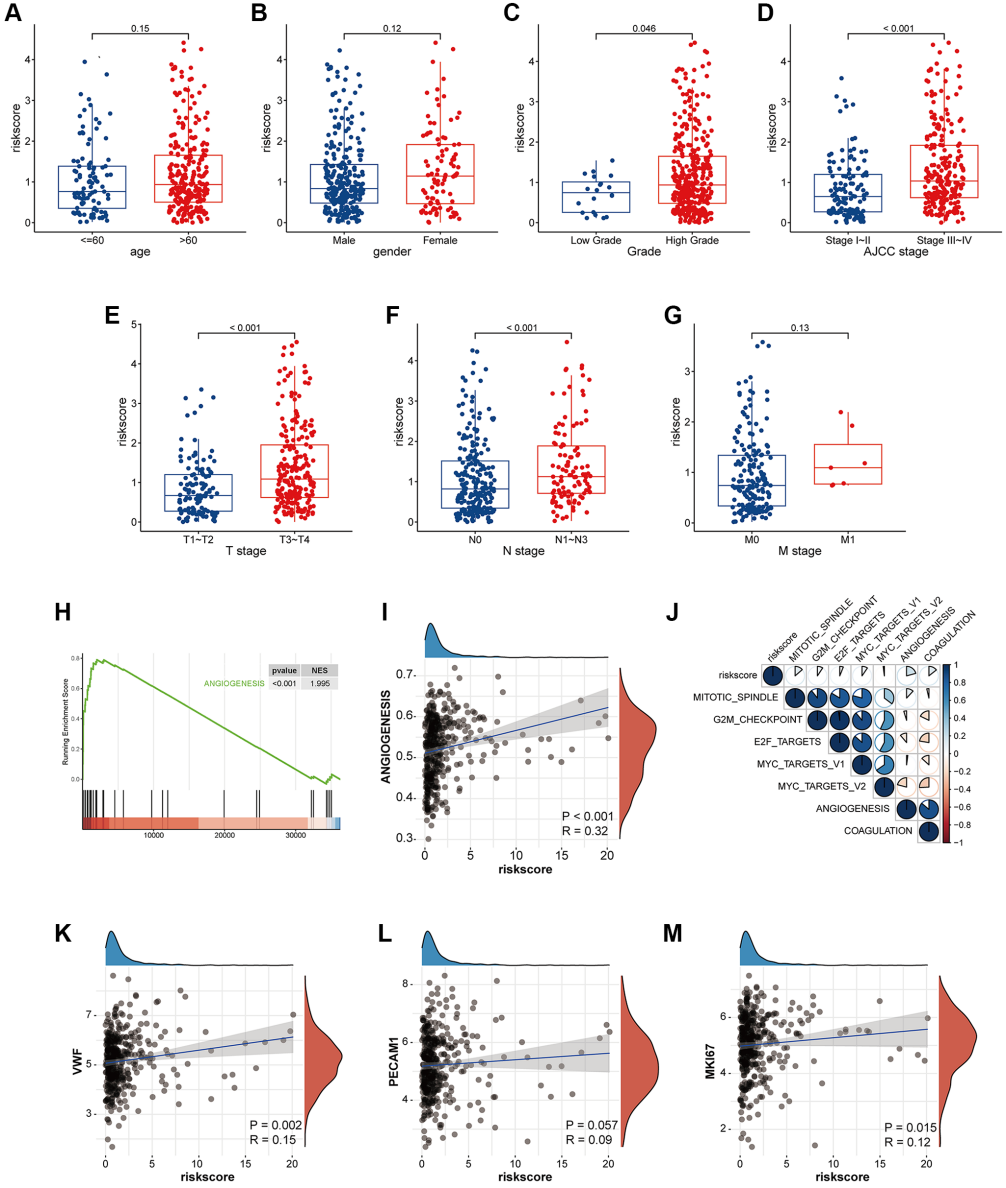
**The risk signature is closely related to tumor proliferation, metastasis, coagulation and angiogenesis.** (**A**–**G**) Box plot of coagulation related risk score by age, gender, pathological grade, AJCC stage, TNM stage. A higher risk score means a higher T, N, AJCC-Stage and a higher grade. The difference was statistically significant. (**H**) GSEA pathways were enriched for angiogenesis based on coagulation-related risk score. NES = 1.995, *p* < 0.001. (**I**) Coagulation-related risk score was strongly positively correlated with angiogenesis. R = 0.32, *p* < 0.001. (**J**–**M**) The coagulation-related risk score was correlated with tumor proliferation, coagulation, and angiogenesis.

To further analyze the correlation between our risk signature and clinical phenotype, we collected clinical information on TCGA bladder tumors and associated phenotypic markers for further analysis. The higher coagulation-related risks were strongly associated with a higher pathological grade of the tumor (*p* = 0.046), a higher clinical stage of AJCC (*p* < 0.001), a higher T stage (*p* < 0.001), and a higher risk of lymph node metastasis (*p* < 0.001) (Figure 4A–4G). The results of the GSEA pathway enrichment analysis suggested that the angiogenic pathways in the tumor were significantly activated (Figure 4H). Pearson correlation analysis suggested that the risk score was strongly correlated with angiogenesis in the tumor (*p* < 0.001, R = 0.32) (Figure 4I). In addition, the risk score was also correlated with tumor coagulation status and cell mitotic cycle (Figure 4J). Finally, we analyzed the correlation between the risk score and the above related molecular markers, and found that the risk score correlated with VWF (*p* = 0.002, R = 0.15), PECAM1 (*p* = 0.057, R = 0.09), and MKI67 (*p* = 0.015, R = 0.12) (Figure 4K–4M).

### Hypercoagulability-related risk signature was closely related to tumor immune microenvironment inhibition

**Figure 5:**
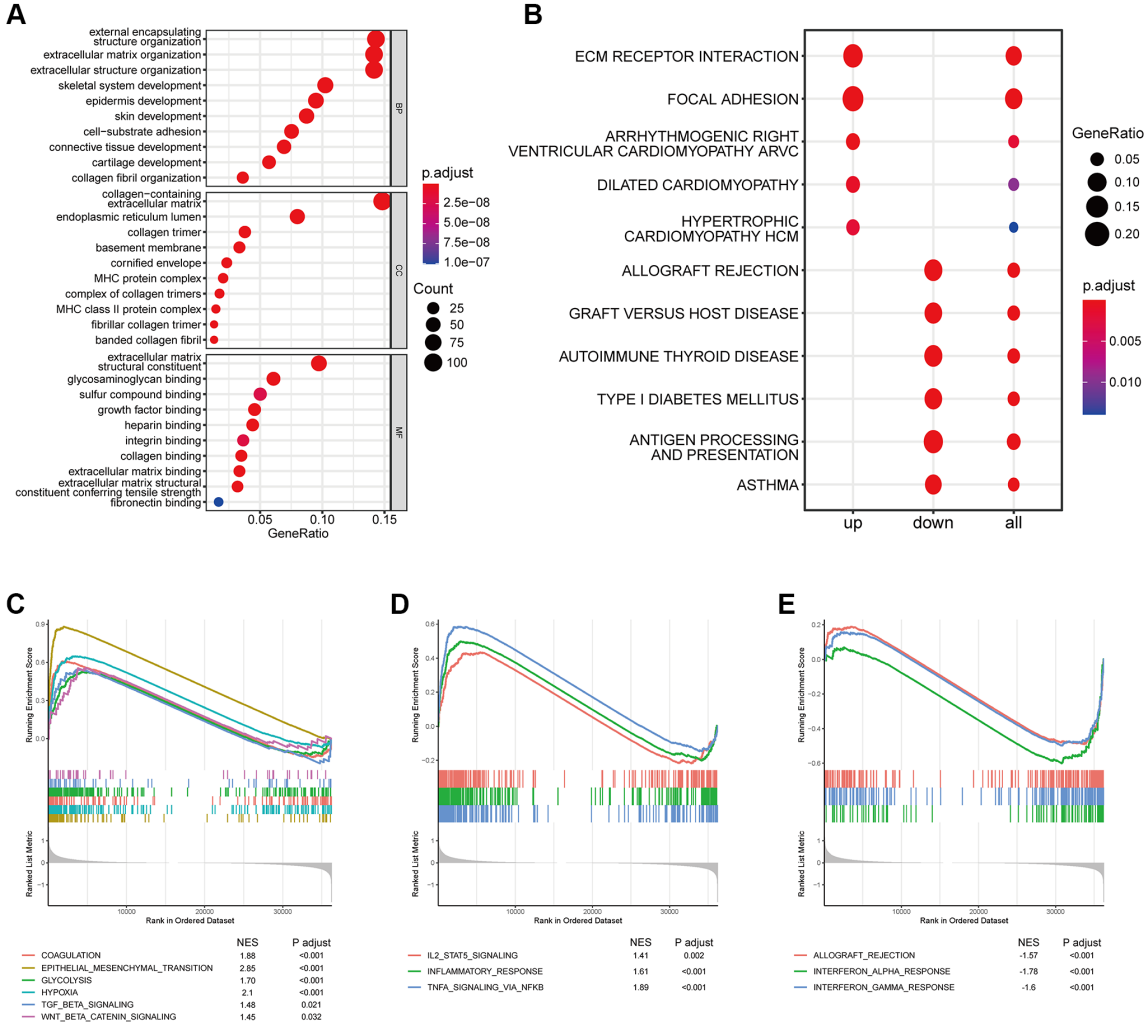
**Effects of abnormal expression of coagulation-related genes on immune-related pathways.** (**A**) GO analysis based on coagulation-related risk signature mainly enriched in the extracellular matrix pathways and MHC pathways. (**B**) KEGG analysis based on coagulation-related risk signature mainly enriched in the ECM receptor interaction pathways. (**C**) GSEA pathways were enriched for coagulation, EMT, hypoxia, WNTb-catenin, TGF-b pathways based on coagulation-related risk signature. (**D**) GSEA pathways were enriched for IL2-STAT, inflammatory response pathways based on coagulation-related risk signature. (**E**) GSEA pathways were enriched for IFNG pathways based on coagulation-related risk signature.

**Figure 6:**
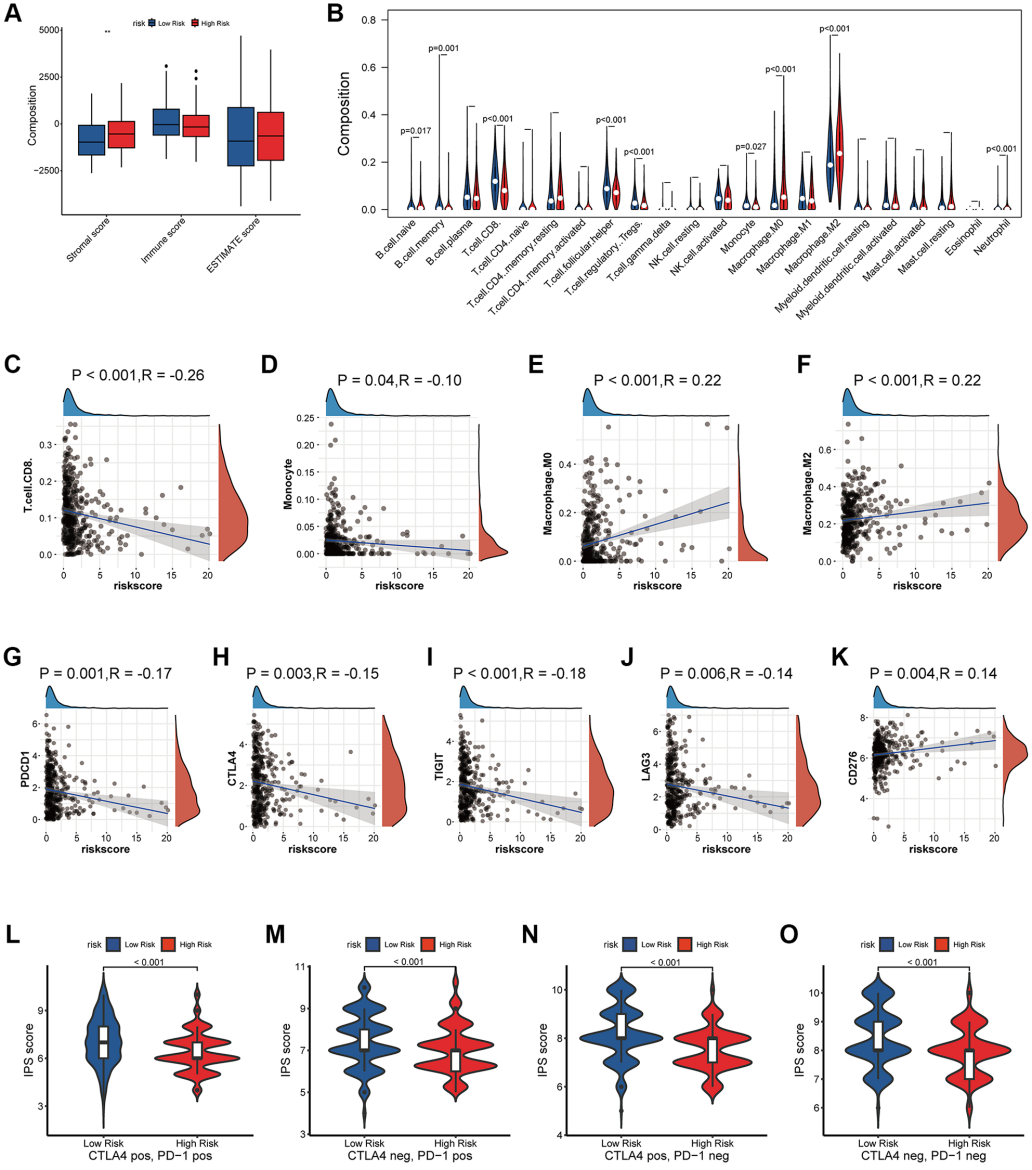
**Effects of abnormal expression of coagulation-related genes on tumor immune microenvironment.** (**A**) Estimate socre analysis based on coagulation-related risk signature. (**B**–**F**) Lymphocyte infiltration analysis based on coagulation-related risk signature. (**G**–**K**) Correlation analysis between coagulation-related risk score and immune checkpoint markers. (**L**–**O**) IPS score analysis based on coagulation-related risk signature.

To further clarify the biological role of the coagulation-related risk signature, we performed GO, KEGG, and GSEA enrichment analyses. The results suggested that the hypercoagulable state not only had a great effect on coagulation-related pathways and proliferation-related pathways that had already been EMT, but also had a great inhibitory effect on the immune microenvironment of tumors (Figure 5, Supplementary Figure 1). For example, TGFβ-β and WNT-β catenin pathways are all activated in hypercoagulable state. The matrix score of the high-risk group was significantly higher than that of the low-risk group, the infiltration abundance of B cells and T cells was significantly lower than that of the risk group, and the infiltration abundance of macrophages was significantly higher than that of the low-risk group. The IGS score and immune related molecular markers also indicated that the low-risk group had significantly better response effects than the high-risk group (Figure 6, Supplementary Figure 2).

### Coagulation-related risk signature predict the efficacy of checkpoint inhibitor therapy for bladder cancer

**Figure 7:**
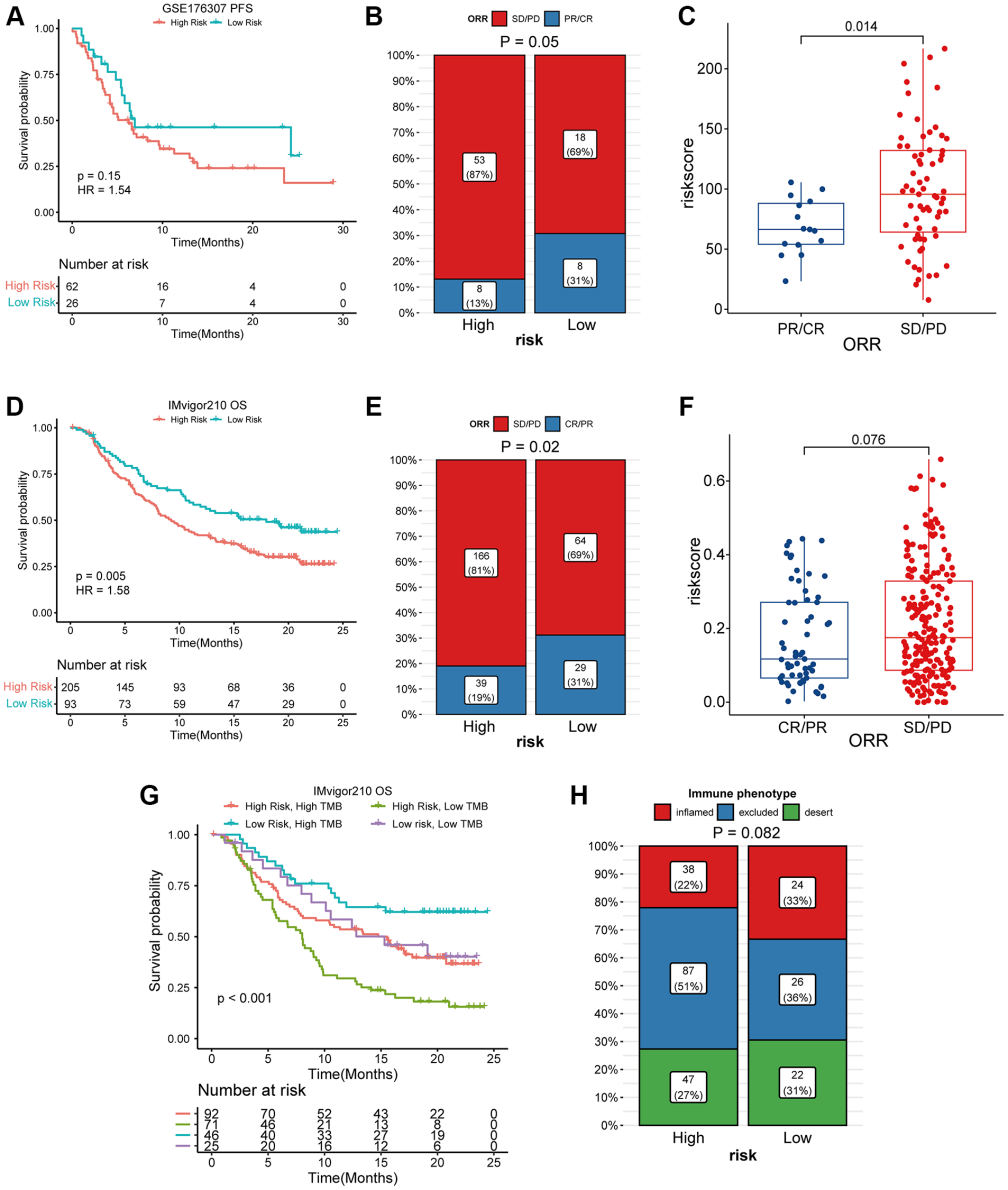
**High coagulation-related risks were associated with poor PD1/PDL1 treatment outcomes and lower responses.** (**A**) K-M curve analysis of coagulation-related risk signature in the GES176307 dataset. (**B**) four-grid table analysis of coagulation-related risk signature in the GES176307 dataset. The sensitivity of immunotherapy was higher in the low-risk group (31% vs. 13%). (**C**) box plot analysis of coagulation-related risk signature in the GES176307 dataset. The risk score was lower in the immunotherapy sensitive group. (**D**) K-M curve analysis of coagulation-related risk signature in the Imvigor210 dataset. (**E**) four-grid table analysis of coagulation-related risk signature in the Imvigor210 dataset. The sensitivity of immunotherapy was higher in the low-risk group (31% vs. 19%). (**F**) box plot analysis of coagulation-related risk signature in the Imvigor210 dataset. The risk score was lower in the immunotherapy sensitive group. (**G**, **H**) K-M curve, four-grid table analysis of TMB combined coagulation-related risk signature in the Imvigor210 dataset.

When the immune microenvironment is in an inhibited state, the therapeutic effect of immune checkpoint inhibitor will be greatly affected [22]. Finally, we validated the predictive power of the risk signature in immunotherapy against two data sets of bladder tumors treated with PD1/PDL1. Our risk signature provides good differentiation in both data sets. In the data set of GSE17637, although the *P* value of KM curve was not statistically significant, it was still considered to have a trend due to the data volume (*p* = 0.15, HR = 1.54). In addition, we found that the high-risk group of bladder cancer patients receiving PD1/PDL1 treatment response was significantly lower than the low-risk group (13% vs. 31%, *p* = 0.05), and for PD1/PDL1 treatment in non- responsive patients the risk score was significantly higher than that in the responsive group (*p* = 0.014) (Figure 7A–7C). In the Imvigor210 data set, the survival prognosis of high-risk group was significantly worse than that of low-risk group (*p* = 0.005, HR = 1.58), the response degree of bladder cancer patients in the high-risk group receiving PD1/PDL1 treatment was significantly lower than that of the low-risk group (19% vs. 31%, *p* = 0.02), and the risk score of patients who failed to respond to PD1/PDL1 treatment was also significantly higher than that of patients in the response group (*p* = 0.076) (Figure 7D–7F). Finally, the four-category result composed of TMB and risk score suggested that risk score could be used as a supplement to TMB (Figure 7G, 7H).

## DISCUSSION

Coagulation abnormalities are very common in advanced tumors, and thromboembolism is the second leading cause of death in patients with advanced tumors, with a incidence rate only lower than organ failure due to distant metastasis of the tumor [23]. About 20% of symptomatic patients with deep vein thrombosis have active malignancies, and the incidence of thrombosis in neoplastic patients is 4–7 times higher than in non-neoplastic patients [24, 25]. Hypercoagulable state in patients with advanced tumor is formed by a combination of many factors. In addition to the factors related to the patients themselves, such as older age and obesity, tumor-related factors are also one of the causes. Studies have confirmed that tumors can secrete inflammatory factors and tumor procoagulant factors activate the clotting mechanism of the body. The cancer cell procoagulant directly promotes the conversion of factor X to factor Xa [26]. The sialic acid portion of the adenocarcinoma mucin causes direct non-enzymatic activation of factor X [27].

Correspondingly, the changes of coagulation state in the body can also affect the biological function of tumors. Coagulation factors are believed to promote cancer invasion and metastasis by promoting the interaction of tumor cell migration and angiogenesis [28]. Neutrophil extracellular traps (NETs) promote tumor cell migration, angiogenesis, and hypercoagulable state [29]. However, whether the abnormal expression of coagulation-related genes affects the biological behavior of tumors is not fully elucidated, and the research on coagulation-related genes in bladder tumors has not been reported yet. In this study, we confirmed the clinical association of coagulation-related genes within tumors with bladder cancer. In large cohort studies, we found that abnormal expression of tumor-associated genes is closely related to the prognosis, angiogenesis, and tumor proliferation of bladder cancer.

Pathway enrichment score indicated that coagulation genes not only activated procoagulant pathways, but also affected angiogenesis, tumor proliferation, and EMT pathways. Tumors need a large amount of energy in the process of growth and invasion, which can be accelerated by angiogenesis and remodeling. The need for angiogenesis in tumor growth and metastasis has been widely recognized [30]. As a symbolic behavior of malignant transformation of cells, EMT has also been widely studied [31]. In addition, our results also suggest that the abnormal expression of coagulation genes may be related to the inhibitory state of the immune microenvironment of bladder cancer. In our study, when the expression of coagulation-related genes was abnormal, immune-related pathways such as TGFβ-β, WNT-β-catenin, and IL6-STAT were all activated. The immune infiltration analysis indicated that when the coagulation related risk score was high, the infiltration abundance of B cells and T cells was low, while that of M2 macrophages was high. In our study, Regulatory T cells (Tregs) were more abundant in the low-risk group. Although considered to be an immunosuppressive cell population, the prognostic role of Tregs varies among tumor types [32]. In bladder cancer, there is a strong positive correlation between Tregs enrichment and T cell infiltration, higher infiltration abundance of Tregs indicates stronger immune cell enrichment and better prognosis [33, 34]. This is consistent with our research results. The state of the immune microenvironment is closely related to the therapeutic efficacy of PD1/PDL1. For example, when the WNT pathway is activated, even highly immunogenic tumor cells will still produce immune escape, thus affecting the therapeutic efficacy of PD1 inhibitors [35]. TGFβ can affect the sensitivity of tumor cells to cytotoxic T cells through a variety of pathways [36, 37]. In our present study, abnormal expression of coagulation genes affected the efficacy of PD1/PDL1 inhibitors for bladder cancer. A high risk associated with coagulation predicts a worse prognosis and a lower response rate.

As a retrospective study, we demonstrated the efficacy of the coagulation-related risk signature in different cohorts, but confounders that could influence clinical outcomes could not be excluded. Although our results confirm that the coagulation score is relevant to the prognosis of bladder cancer and the therapeutic efficacy of PD1/PDL1, further studies are needed to confirm the clinical utility of the coagulation score and its utility as a useful biomarker.

In conclusion, we provide a method for quantitative assessment of coagulation status within a tumor. At the same time, coagulation scores were strongly associated with angiogenesis, tumor proliferation, EMT, and poor prognosis in patients with bladder cancer as well as in those treated with PD1/PDL1.

## Supplementary Materials

Supplementary Figures

## References

[1] Kim AS, Khorana AA, McCrae KR. Mechanisms and biomarkers of cancer-associated thrombosis. Transl Res. 2020; 225:33–53. 10.1016/j.trsl.2020.06.012 32645431 PMC8020882

[2] Labelle M, Begum S, Hynes RO. Direct signaling between platelets and cancer cells induces an epithelial-mesenchymal-like transition and promotes metastasis. Cancer Cell. 2011; 20:576–90. 10.1016/j.ccr.2011.09.009 22094253 PMC3487108

[3] Parisi R, Panzera T, Russo L, Gamba S, De Curtis A, Di Castelnuovo A, Marchetti M, Cerletti C, Falanga A, de Gaetano G, Donati MB, Iacoviello L, Costanzo S, and Moli-sani Study Investigators. Fibrinogen levels in relation to colorectal cancer onset: A nested case-cohort study from the Moli-sani cohort. Front Cardiovasc Med. 2022; 9:1009926. 10.3389/fcvm.2022.1009926 36312278 PMC9606318

[4] Henrikson KP, Salazar SL, Fenton JW 2nd, Pentecost BT. Role of thrombin receptor in breast cancer invasiveness. Br J Cancer. 1999; 79:401–6. 10.1038/sj.bjc.6690063 10027305 PMC2362433

[5] Diao X, Guo C, Li S. Identification of a novel anoikis-related gene signature to predict prognosis and tumor microenvironment in lung adenocarcinoma. Thorac Cancer. 2023; 14:320–30. 10.1111/1759-7714.14766 36507553 PMC9870742

[6] Wang Y, Lin MG, Meng L, Chen ZM, Wei ZJ, Ying SC, Xu A. Identification of necroptosis-related genes for predicting prognosis and exploring immune infiltration landscape in colon adenocarcinoma. Front Oncol. 2022; 12:941156. 10.3389/fonc.2022.941156 36505813 PMC9731216

[7] Xu R, Wu X, Du A, Zhao Q, Huang H. Identification of cuproptosis-related long non-coding ribonucleic acid signature as a novel prognosis model for colon cancer. Am J Cancer Res. 2022; 12:5241–54. 36504883 PMC9729908

[8] Colaprico A, Silva TC, Olsen C, Garofano L, Cava C, Garolini D, Sabedot TS, Malta TM, Pagnotta SM, Castiglioni I, Ceccarelli M, Bontempi G, Noushmehr H. TCGAbiolinks: an R/Bioconductor package for integrative analysis of TCGA data. Nucleic Acids Res. 2016; 44:e71. 10.1093/nar/gkv1507 26704973 PMC4856967

[9] Lee JS, Leem SH, Lee SY, Kim SC, Park ES, Kim SB, Kim SK, Kim YJ, Kim WJ, Chu IS. Expression signature of E2F1 and its associated genes predict superficial to invasive progression of bladder tumors. J Clin Oncol. 2010; 28:2660–7. 10.1200/JCO.2009.25.0977 20421545

[10] Mariathasan S, Turley SJ, Nickles D, Castiglioni A, Yuen K, Wang Y, Kadel EE III, Koeppen H, Astarita JL, Cubas R, Jhunjhunwala S, Banchereau R, Yang Y, et al. TGFβ attenuates tumour response to PD-L1 blockade by contributing to exclusion of T cells. Nature. 2018; 554:544–8. 10.1038/nature25501 29443960 PMC6028240

[11] Rose TL, Weir WH, Mayhew GM, Shibata Y, Eulitt P, Uronis JM, Zhou M, Nielsen M, Smith AB, Woods M, Hayward MC, Salazar AH, Milowsky MI, et al. Fibroblast growth factor receptor 3 alterations and response to immune checkpoint inhibition in metastatic urothelial cancer: a real world experience. Br J Cancer. 2021; 125:1251–60. 10.1038/s41416-021-01488-6 34294892 PMC8548561

[12] Wagner GP, Kin K, Lynch VJ. Measurement of mRNA abundance using RNA-seq data: RPKM measure is inconsistent among samples. Theory Biosci. 2012; 131:281–5. 10.1007/s12064-012-0162-3 22872506

[13] Monti S, Tamayo P, Mesirov J, Golub T. Consensus Clustering: A Resampling-Based Method for Class Discovery and Visualization of Gene Expression Microarray Data. Mach Learn. 2003; 52:91–118. 10.1023/A:1023949509487

[14] Ritchie ME, Phipson B, Wu D, Hu Y, Law CW, Shi W, Smyth GK. limma powers differential expression analyses for RNA-sequencing and microarray studies. Nucleic Acids Res. 2015; 43:e47. 10.1093/nar/gkv007 25605792 PMC4402510

[15] Friedman J, Hastie T, Tibshirani R. Regularization Paths for Generalized Linear Models via Coordinate Descent. J Stat Softw. 2010; 33:1–22. 20808728 PMC2929880

[16] Barros AJ, Hirakata VN. Alternatives for logistic regression in cross-sectional studies: an empirical comparison of models that directly estimate the prevalence ratio. BMC Med Res Methodol. 2003; 3:21. 10.1186/1471-2288-3-21 14567763 PMC521200

[17] Huang ML, Hung YH, Lee WM, Li RK, Jiang BR. SVM-RFE based feature selection and Taguchi parameters optimization for multiclass SVM classifier. ScientificWorldJournal. 2014; 2014:795624. 10.1155/2014/795624 25295306 PMC4175386

[18] Robin X, Turck N, Hainard A, Tiberti N, Lisacek F, Sanchez JC, Müller M. pROC: an open-source package for R and S+ to analyze and compare ROC curves. BMC Bioinformatics. 2011; 12:77. 10.1186/1471-2105-12-77 21414208 PMC3068975

[19] Zhou RS, Zhang EX, Sun QF, Ye ZJ, Liu JW, Zhou DH, Tang Y. Integrated analysis of lncRNA-miRNA-mRNA ceRNA network in squamous cell carcinoma of tongue. BMC Cancer. 2019; 19:779. 10.1186/s12885-019-5983-8 31391008 PMC6686570

[20] Newman AM, Liu CL, Green MR, Gentles AJ, Feng W, Xu Y, Hoang CD, Diehn M, Alizadeh AA. Robust enumeration of cell subsets from tissue expression profiles. Nat Methods. 2015; 12:453–7. 10.1038/nmeth.3337 25822800 PMC4739640

[21] Yu G, Wang LG, Han Y, He QY. clusterProfiler: an R package for comparing biological themes among gene clusters. OMICS. 2012; 16:284–7. 10.1089/omi.2011.0118 22455463 PMC3339379

[22] Wang D, Ye Q, Gu H, Chen Z. The role of lipid metabolism in tumor immune microenvironment and potential therapeutic strategies. Front Oncol. 2022; 12:984560. 10.3389/fonc.2022.984560 36172157 PMC9510836

[23] Elyamany G, Alzahrani AM, Bukhary E. Cancer-associated thrombosis: an overview. Clin Med Insights Oncol. 2014; 8:129–37. 10.4137/CMO.S18991 25520567 PMC4259501

[24] Donnellan E, Kevane B, Bird BR, Ainle FN. Cancer and venous thromboembolic disease: from molecular mechanisms to clinical management. Curr Oncol. 2014; 21:134–43. 10.3747/co.21.1864 24940094 PMC4059798

[25] Lee AY. Management of thrombosis in cancer: primary prevention and secondary prophylaxis. Br J Haematol. 2005; 128:291–302. 10.1111/j.1365-2141.2004.05292.x 15667530

[26] Falanga A, Gordon SG. Isolation and characterization of cancer procoagulant: a cysteine proteinase from malignant tissue. Biochemistry. 1985; 24:5558–67. 10.1021/bi00341a041 3935163

[27] Bick RL. Cancer-associated thrombosis. N Engl J Med. 2003; 349:109–11. 10.1056/NEJMp030086 12853582

[28] Wojtukiewicz MZ, Hempel D, Sierko E, Tucker SC, Honn KV. Protease-activated receptors (PARs)--biology and role in cancer invasion and metastasis. Cancer Metastasis Rev. 2015; 34:775–96. 10.1007/s10555-015-9599-4 26573921 PMC4661218

[29] Efrimescu CI, Buggy PM, Buggy DJ. Neutrophil Extracellular Trapping Role in Cancer, Metastases, and Cancer-Related Thrombosis: a Narrative Review of the Current Evidence Base. Curr Oncol Rep. 2021; 23:118. 10.1007/s11912-021-01103-0 34342735 PMC8330188

[30] Katsuta E, Rashid OM, Takabe K. Clinical relevance of tumor microenvironment: immune cells, vessels, and mouse models. Hum Cell. 2020; 33:930–7. 10.1007/s13577-020-00380-4 32507979

[31] Pastushenko I, Blanpain C. EMT Transition States during Tumor Progression and Metastasis. Trends Cell Biol. 2019; 29:212–26. 10.1016/j.tcb.2018.12.001 30594349

[32] Shang B, Liu Y, Jiang SJ, Liu Y. Prognostic value of tumor-infiltrating FoxP3+ regulatory T cells in cancers: a systematic review and meta-analysis. Sci Rep. 2015; 5:15179. 10.1038/srep15179 26462617 PMC4604472

[33] Leblond MM, Zdimerova H, Desponds E, Verdeil G. Tumor-Associated Macrophages in Bladder Cancer: Biological Role, Impact on Therapeutic Response and Perspectives for Immunotherapy. Cancers (Basel). 2021; 13:4712. 10.3390/cancers13184712 34572939 PMC8467100

[34] Koll FJ, Banek S, Kluth L, Köllermann J, Bankov K, Chun FK, Wild PJ, Weigert A, Reis H. Tumor-associated macrophages and Tregs influence and represent immune cell infiltration of muscle-invasive bladder cancer and predict prognosis. J Transl Med. 2023; 21:124. 10.1186/s12967-023-03949-3 36793050 PMC9930232

[35] Takeuchi Y, Tanegashima T, Sato E, Irie T, Sai A, Itahashi K, Kumagai S, Tada Y, Togashi Y, Koyama S, Akbay EA, Karasaki T, Kataoka K, et al. Highly immunogenic cancer cells require activation of the WNT pathway for immunological escape. Sci Immunol. 2021; 6:eabc6424. 10.1126/sciimmunol.abc6424 34767457

[36] Bagati A, Kumar S, Jiang P, Pyrdol J, Zou AE, Godicelj A, Mathewson ND, Cartwright ANR, Cejas P, Brown M, Giobbie-Hurder A, Dillon D, Agudo J, et al. Integrin αvβ6-TGFβ-SOX4 Pathway Drives Immune Evasion in Triple-Negative Breast Cancer. Cancer Cell. 2021; 39:54–67.e9. 10.1016/j.ccell.2020.12.001 33385331 PMC7855651

[37] Larson C, Oronsky B, Carter CA, Oronsky A, Knox SJ, Sher D, Reid TR. TGF-beta: a master immune regulator. Expert Opin Ther Targets. 2020; 24:427–38. 10.1080/14728222.2020.1744568 32228232

