# Supplementary material for: Procoagulant genes may affect angiogenesis, epithelial-mesenchymal transition, survival prognosis and tumor immune microenvironment in patients with urothelial carcinoma
Source: Aging (Albany NY). 2023 Jul 8;15(13):6429–44. doi: 10.18632/aging.204860 (PMC10373971; doi:10.18632/aging.204860)
Supplement: Supplementary Figures [file aging-15-204860-s001.pdf]

SUPPLEMENTARY FIGURES

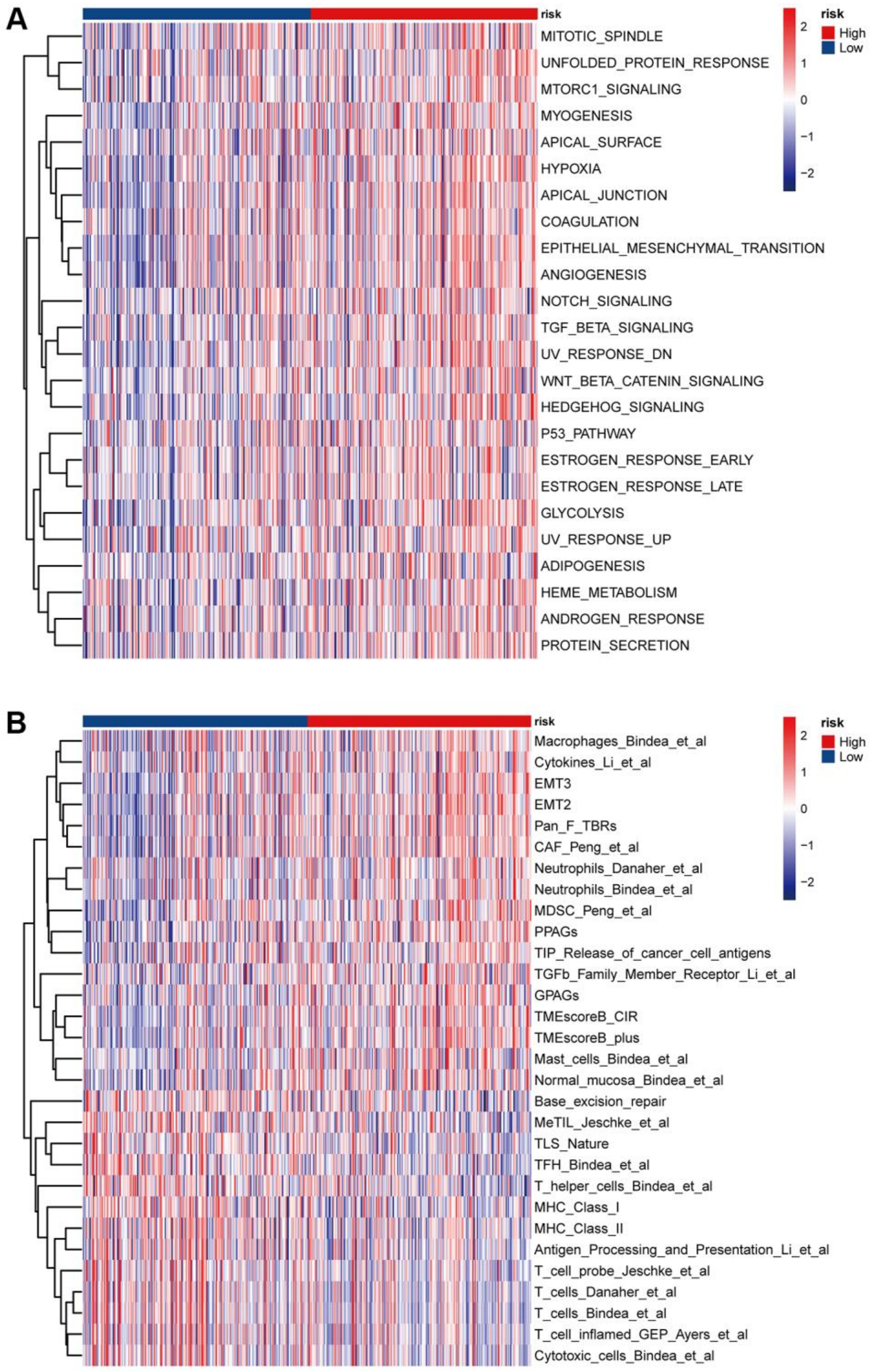

Supplementary Figure 1. ssGSEA heatmap based on coagulation-related risk signature. (**A**) The distribution of high-risk and low-risk groups in metabolic related pathways analysed by ssGSEA. (**B**) The distribution of high-risk and low-risk groups in tumor immune microenvironment related pathways analysed by ssGSEA.

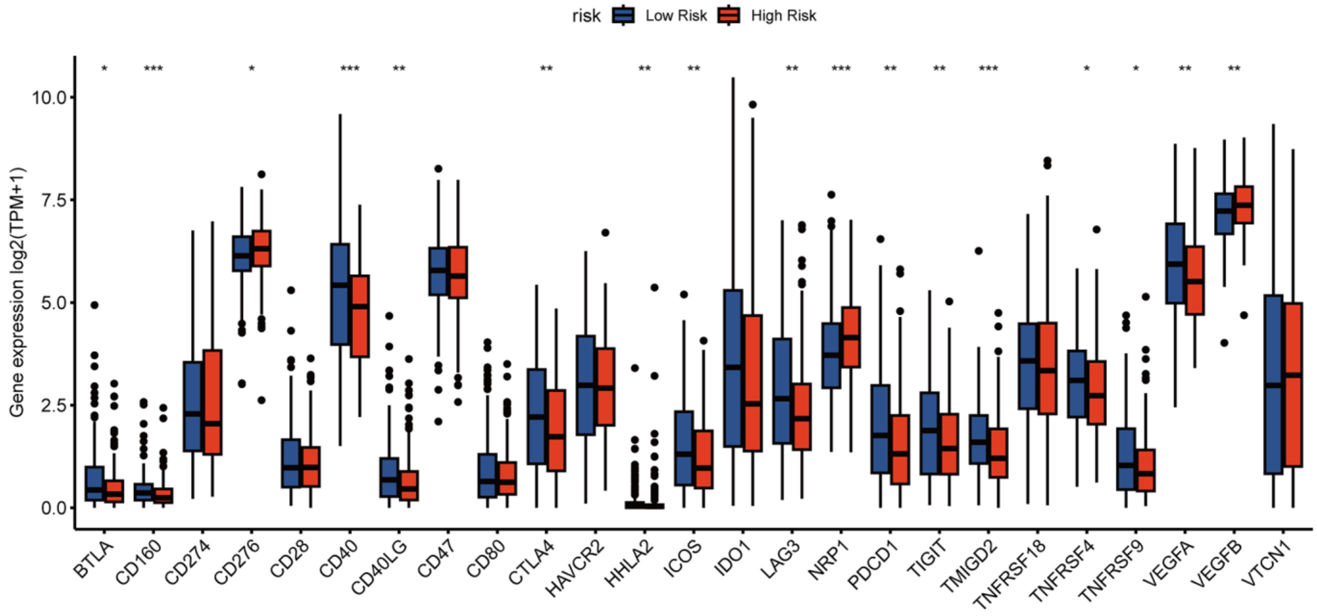

Supplementary Figure 2. Box diagram analysis of immune related markers.
